# Crystal structure of *Helicobacter pylori* MinE, a cell division topological specificity factor

**DOI:** 10.1111/j.1365-2958.2010.07160.x

**Published:** 2010-04-27

**Authors:** Gil Bu Kang, Hye-Eun Song, Mun-Kyoung Kim, Hyung-Seop Youn, Jung-Gyu Lee, June Yop An, Jang-Soo Chun, Hyesung Jeon, Soo Hyun Eom

**Affiliations:** 1 School of Life Science, Gwangju Institute of Science & Technology (GIST)Gwangju 500-712, Korea; 2 Biomedical Research Center, Korea Institute of Science and TechnologySeoul 136-791, Korea

## Abstract

In Gram-negative bacteria, proper placement of the FtsZ ring, mediated by nucleoid occlusion and the activities of the dynamic oscillating Min proteins MinC, MinD and MinE, is required for correct positioning of the cell division septum. MinE is a topological specificity factor that counters the activity of MinCD division inhibitor at the mid-cell division site. Its structure consists of an anti-MinCD domain and a topology specificity domain (TSD). Previous NMR analysis of truncated *Escherichia coli* MinE showed that the TSD domain contains a long α-helix and two anti-parallel β-strands, which mediate formation of a homodimeric α/β structure. Here we report the crystal structure of full-length *Helicobacter pylori* MinE and redefine its TSD based on that structure. The N-terminal region of the TSD (residues 19–26), previously defined as part of the anti-MinCD domain, forms a β-strand (βA) and participates in TSD folding. In addition, *H. pylori* MinE forms a dimer through the interaction of anti-parallel βA-strands. Moreover, we observed serial dimer–dimer interactions within the crystal packing, resulting in the formation of a multimeric structure. We therefore redefine the functional domain of MinE and propose that a multimeric filamentous structure is formed through anti-parallel β-strand interactions.

## Introduction

In most organisms, cell division occurs after placement of a septum through the midpoint of the dividing cell and equal distribution of the cellular components into the two daughter cells (Rothfield *et al*., 2005). This process is driven by the formation of a FtsZ ring at the division site. The rod-shaped bacteria *Escherichia coli* (Gram-negative) and *Bacillus subtilis* (Gram-positive) and the curved *Caulobacter crescentus*, which is representative of very different species, all have been extensively used as models in the study of cell morphogenesis (Lutkenhaus, 2007). In Gram-negative bacteria, including *E. coli*, proper placement of the FtsZ ring is mediated by nucleoid occlusion and activities of the dynamic oscillating Min proteins MinC, MinD and MinE (RayChaudhuri *et al*., 2000; Møller-Jensen and Löwe, 2005; Gitai, 2007), which act in concert to prevent septation at sites other than the mid-cell region. MinD is a membrane assembly protein responsible for recruiting MinC and MinE to the membrane. MinC and MinE appear to be located in the cytoplasm when expressed in the absence of MinD, but become membrane-associated when coexpressed with MinD (de Boer *et al*., 1991; Hayashi *et al*., 2001; Ma *et al*., 2003; Szeto *et al*., 2005). MinC is a division inhibitor that binds to FtsZ and inhibits the formation of a stable FtsZ ring, which is necessary for the division process. However, MinC lacks site specificity; consequently, when expressed in the absence of MinD and MinE, MinC mediates a global block of cell division, resulting in the formation of long filamentous cells (de Boer *et al*., 1989; Cordell *et al*., 2001). MinE induces the redistribution of MinC and MinD such that a membrane-associated polar zone containing MinC, MinD and MinE is formed at one end of the cell (Raskin and de Boer, 1997; Fu *et al*., 2001; Rothfield *et al*., 2001). MinE then assembles into a ring-like structure (E-ring) near mid-cell. Subsequently, the E-ring and polar zone undergo disassembly and then reassembly at the opposite pole of the cell. This oscillation of Min proteins from pole to pole occurs many times in each division cycle (Hale *et al*., 2001; Hu and Lutkenhaus, 2001). As a result of this repeated cycle, the time-averaged concentration of the MinC division inhibitor is lowest near mid-cell, which enables assembly of a septum at this site.

MinE has two structurally and functionally distinct domains: the anti-MinCD domain and the topological specificity domain (TSD) (Pichoff *et al*., 1995; Zhao *et al*., 1995). The TSD (residues 32–88) of *E. coli* MinE (*Ec*MinE) is required for E-ring formation and for formation of the polar zones. An earlier NMR study using truncated *Ec*MinE (residues 32–88) showed that MinE has a homodimeric α/β structure and that each monomer contains a long α-helix and an anti-parallel β-hairpin, which, together with two C-terminal β-strands, forms a four-stranded anti-parallel β-sheet (King *et al*., 2000). In contrast, the anti-MinCD domain of *Ec*MinE (residues 1–31) interacts with MinD and induces the dissociation of the MinCD complex, thereby blocking the inhibitory action of MinC on cell division. It has been proposed that the anti-MinCD domain assumes an α-helical conformation. In addition, secondary chemical shift analysis of full-length MinE from *Neisseria gonorrhoeae* (*Ng*MinE) using NMR showed that the C-terminal portion of the anti-MinCD domain assumes a β-conformation (residues 21–30) and that there is also an N-terminal helix (residues 3–8) (Ramos *et al*., 2006). Because of a lack of structural information, however, the functions of MinE, including the interaction of MinE with MinD to inhibit the MinCD complex and MinE ring formation, are well not understood.

Here we report the crystal structure of full-length *Helicobacter pylori* MinE (*Hp*MinE) and redefine the TSD of *Hp*MinE based on that structure. Remarkably, we found that the N-terminal region of the TSD (residues 19–26), which was previously defined as part of the anti-MinCD domain, forms a β-strand (βA) that participates in TSD folding, and *Hp*MinE forms a dimer through the interaction of anti-parallel βA-strands. Within the crystal packing, we observed a multimeric structure formed by serial dimer–dimer interactions.

## Results and discussion

### Overall structure of *Hp*MinE

**Fig. 1:**
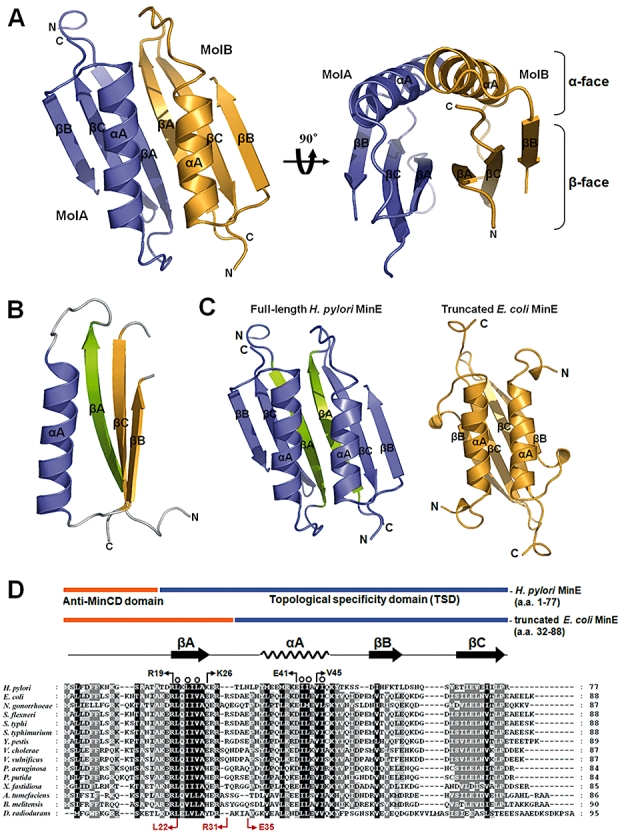
Crystal structure and sequence alignment of full-length *Hp*MinE. A. Ribbon view showing the overall dimeric structure of *Hp*MinE. Subunits MolA and MolB are coloured blue and orange respectively. The homodimeric interface of *Hp*MinE is comprised of the α-face (the anti-parallel α-helices) and the β-face (six anti-parallel β-strands). These figures were made using PyMOL (DeLano, 2002). B. Ribbon view of the *Hp*MinE monomer. The monomeric structure consists of an α-helix (αA) and three anti-parallel β-strands (βA, βB and βC). Helix αA and two β-strands (βB and βC) are coloured blue and orange respectively. The β-strand located at the dimer interface (βA) is coloured green. C. Comparison of the structures of the full-length *Hp*MinE and truncated *Ec*MinE (pdb id 1EV0) dimers. Full-length *Hp*MinE is shown in blue, except the N-terminal strand (βA) is shown in green. Truncated *Ec*MinE is shown in orange. D. Sequence alignment of *Hp*MinE with homologous sequences from other Gram-negative bacteria. The amino acid sequences of MinE from *H. pylori*, *E. coli*, *N. gonorrhoeae*, *Shigella flexneri*, *Salmonella typhi*, *Salmonella typhimurium*, *Yersinia pestis*, *Vibrio cholerae*, *Vibrio vulnificus*, *Pseudomonas aeruginosa*, *Pseudomonas putida*, *Xylella fastidiosa*, *Agrobacterium tumefaciens*, *Brucella melitensis* and *Deinococcus radiodurans* were aligned using the Clustal X programme (Thompson *et al*., 1997). The locations of the anti-MinCD domain (orange) and TSD (blue) in the *Hp*MinE are indicated. Residues that contribute to the hydrophobic core between the α-face and β-face of the *Hp*MinE dimer are indicated by open circles above sequences.

The crystal structure of full-length *Hp*MinE was solved using the multi-wavelength anomalous dispersion (MAD) method and refined to an *R*_work_ = 26.2% and *R*_free_ = 29.6% at 2.8 Å resolution. Two monomers were observed in an asymmetric unit; they formed a homodimeric α/β structure with an upper surface (the α-face) consisting of α-helix and a lower surface (the β-face) consisting of an anti-parallel twisted β-sheet (Fig. 1A). The extensive hydrophobic dimer interface subsumed about 1100 Å^2^ of surface area (22.8% of the total surface area), and size exclusion chromatography showed that *Hp*MinE forms a stable dimer in solution (data not shown). Each monomer is composed of a long helix αA (residues 35–50) and a three-stranded anti-parallel β-sheet comprised of βA (residues 19–26), βB (residues 54–59) and βC (residues 67–74) (Fig. 1B). The N-terminus of *Hp*MinE (residues 1–12 in MolA; residues 1–15 in MolB) was disordered and invisible within the crystal structure.

### Structure of the *Hp*MinE monomer

MinE contains an N-terminal anti-MinCD domain and a C-terminal TSD (Pichoff *et al*., 1995; Zhao *et al*., 1995). The anti-MinCD domain was previously defined as being comprised of residues 1–31 in *Ec*MinE (residues 1–28 in *Hp*MinE) and was predicted to assume an α-helical structure. Consistent with that idea, NMR studies showed that the N-terminus of the anti-MinCD domain, corresponding to residues 1–22 in *Ec*MinE (residues 1–20 in *Hp*MinE), exists as a nascent α-helix in solution (King *et al*., 2000). In the case of *Ng*MinE, only residues 3–8 form an α-helix (residues 3–8 in *Hp*MinE), while the C-terminal portion (residues 21–30) of the anti-MinCD domain forms a β-strand (residues 19–28 in *Hp*MinE) (Ramos *et al*., 2006). The TSD was previously defined as being comprised of residues 32–88 in *Ec*MinE (residues 29–77 in *Hp*MinE). King *et al*. (2000) reported that the NMR structure of the TSD in *Ec*MinE contains a long α-helix (αA) and two anti-parallel β-strands (βB and βC), which form a homodimeric α/β structure through the interaction of anti-parallel βC stands (Fig. 1C).

Consistent with NMR analysis of *Ng*MinE, the N-terminal region (residues 19–26, previously defined as part of the anti-MinCD domain) of the full-length *Hp*MinE forms strand βA. To our surprise, however, strand βA participates in the folding of the TSD, and strands βA, βC and βB form a continuous three-stranded anti-parallel β-sheet within the TSD. Based on that structure, we redefine the TSD of *Hp*MinE and suggest it is responsible for the formation of the E-ring [residues 19–77, consisting of helix αA and three β-strands (βA, βB and βC)]. Moreover, the sequence of the strand βA region of *Hp*MinE is highly conserved among Gram-negative bacterial MinEs (Fig. 1D). This suggests it is likely that the newly defined TSD, as well as strand βA within in the N-terminal portion of the TSD in *Hp*MinE, can be applied to most rod-shaped, Gram-negative bacteria, including *E. coli*.

### The novel dimer interface within *Hp*MinE

**Fig. 2:**
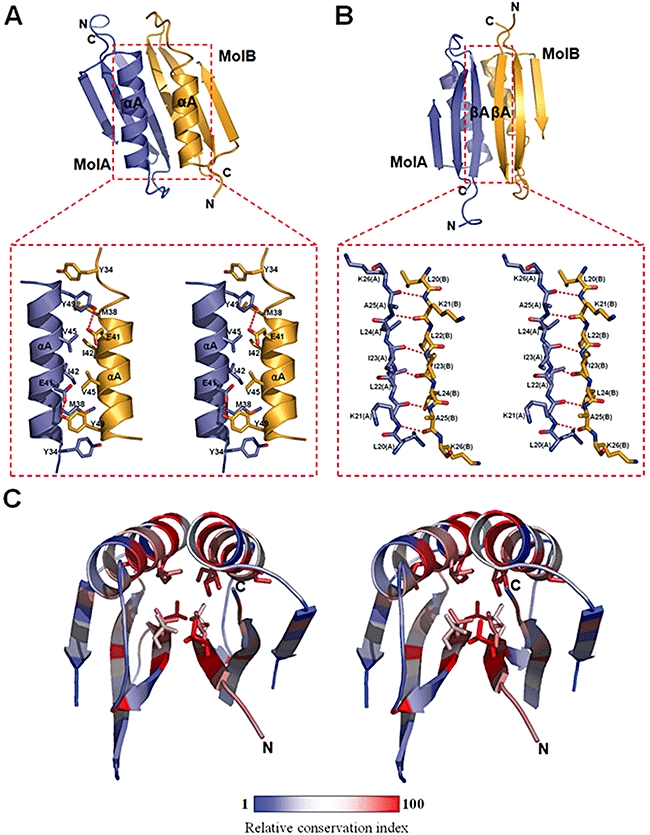
The dimer interface within the *Hp*MinE structure. A. Close-up view of the interacting residues in the α-face (Y34, M38, E41, I42, V45 and Y49). Protomers MolA and MolB are coloured blue and orange respectively. The four-amino acid cluster comprised of E41 and V45 from each subunit is located at the centre of the α-face. In particular, the E41 residues (MolA/MolB) make hydrogen bonds with the respective Y49 residues. Hydrogen bonds are shown as dotted lines. B. Close-up view of the interacting residues in the β-face (L20, K21, L22, I23, L24 and A25). Hydrogen bonds are shown as dotted lines. C. The dimeric structure of *Hp*MinE coloured according to a relative conservation index (1 to 100) based on homologous MinE sequences from other Gram-negative bacteria, including *E. coli*, *N. gonorrhoeae*, *S. flexneri*, *S. typhi*, *S. typhimurium*, *Y. pestis*, *V. cholerae*, *V. vulnificus*, *P. aeruginosa*, *P. putida*, *X. fastidiosa*, *A. tumefaciens*, *B. melitensis* and *D. radiodurans*. The relative conservation index was calculated using the Clustal X programme. The residues that form the hydrophobic core between the α-face and β-face (L20, L22, L24, I42, I43 and I46) are highly conserved.

The structure of the *Hp*MinE dimer shows a distinct interface between two monomers accompanied by an anti-parallel arrangement of the long helix αA (α-face) and strand βA (β-face). Within the α-face, the dimer interface is formed by two anti-parallel coiled coils, which participate in hydrophobic interactions through the highly conserved hydrophobic residues Y34, M38, I42, V45 and Y49 (Fig. 2A). In addition, the hydrophobic interaction is strengthened by two hydrogen bonds between E41 (MolA/MolB) and Y49 (MolB/MolA). The E41 and Y49 residues are conserved among Gram-negative bacteria as E/D and Y/H residues, indicating that the hydrogen bonds stabilizing the hydrophobic interface are also conserved.

In the β-face of the dimer structure, the first N-terminal strand βA of each monomer, which corresponds to residues 21–28 in *Ec*MinE, make anti-parallel β-strands, and this interaction results in the formation of a contiguous anti-parallel twisted β-sheet (Fig. 2B). This β-sheet packs against the anti-parallel α-helices, forming the hydrophobic core between the α-face and the β-face. The hydrophobic residues L20, L22, L24, I42, I43 and I46, buried in the hydrophobic core, are highly conserved among Gram-negative bacterial MinEs (Fig. 2C). It is noteworthy that the *Ec*MinE structure, which lacks the N-terminal strand βA within its TSD, shows an anti-parallel β-strand interaction made by strand βC, which would seem unnatural (Fig. 1C). In order to confirm the structural function of strand βA in the dimerization seen in the crystal structure, we prepared N-terminal deletion mutants and analysed the mutational effects on multimerization using analytical gel filtration chromatography. Two deletion mutants lacking strand βA [residues 32–88 in *Ec*MinE (*Ec*MinEΔN) and 29-77 in *Hp*MinE (*Hp*MinEΔN)] were designed, and the results of the analytical gel filtration are summarized in Table S1. At a concentration of 0.5 mM, which is the concentration used for the gel filtration analysis, about 86% of *Ec*MinE existed as dimer, as calculated from the dissociation constants of the monomer–dimer and dimer–tetramer equilibriums obtained in equilibrium sedimentation experiments (Zhang *et al*., 1998). Consistent with that finding, we observed that *Ec*MinE and *Hp*MinE behaved as dimers, although they eluted a little earlier than the expected dimer fraction, probably due to the flexible N-terminal anti-MinCD domain. By contrast, *Ec*MinEΔN and *Hp*MinEΔN were eluted as a tetramer and trimer respectively. The importance of the N-terminal region (residues 22–35, containing the strand βA region) of *Ec*MinE in dimerization was also apparent in studies of hetero-oligomer formation using non-denaturing PAGE (Zhang *et al*., 1998). Collectively, these findings show that strand βA in the N-terminal region is critical for proper dimer formation, and we expect that the dimeric structure formed by the anti-parallel strand βA interaction observed in the crystal structure represents the physiological dimer structure.

**Fig. 3:**
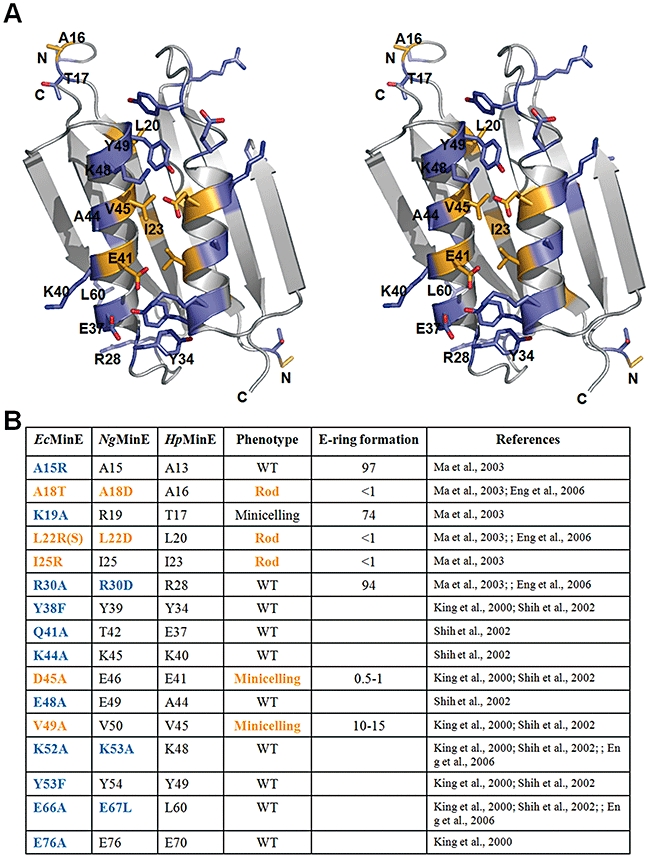
MinE residues important for topological specificity function and E-ring formation. A. Representation of *Hp*MinE residues that correspond to mutated *Ec*MinE or *Ng*MinE residues used to determine which residues are important for topological specificity function and E-ring formation. Residues important for topological specificity and E-ring formation are coloured orange; less important residues are coloured blue. B. Summary of the effects of MinE mutants on the cell division phenotype and E-ring formation. E-ring formation indicates the percentage of cells with E-rings. Phenotype is classified into three distinct types based on the cell morphology. WT, wild-type phenotype; Rod, filamentous phenotype; Minicelling, minicelling phenotype containing a mixed population of minicells, wild-type cells and short filaments.

It was previously reported that various MinE mutations affect the cell division phenotype and E-ring formation (King *et al*., 2000; Shih *et al*., 2002; Ma *et al*., 2003; Eng *et al*., 2006). Among such mutations, the A18T, L22R, I25R, D45A and V49A *Ec*MinE mutants (A16, L20, I23, E41 and V45 in *Hp*MinE) lost topological specificity and prevented E-ring formation. Interestingly, all of the corresponding residues in *Hp*MinE, except A16, are located at the dimer interface and are involved in the dimer interaction (Fig. 3). This implies the mutants may weaken the dimer interaction, which would result in a loss of topological specificity, causing a defect in the formation or stability of the E-ring. We therefore suggest that the dimer interaction of MinE is critical for the assembly of multimeric structures and eventually for topological specificity and E-ring formation.

### Dimer–dimer interactions in the *Hp*MinE structure

**Fig. 4:**
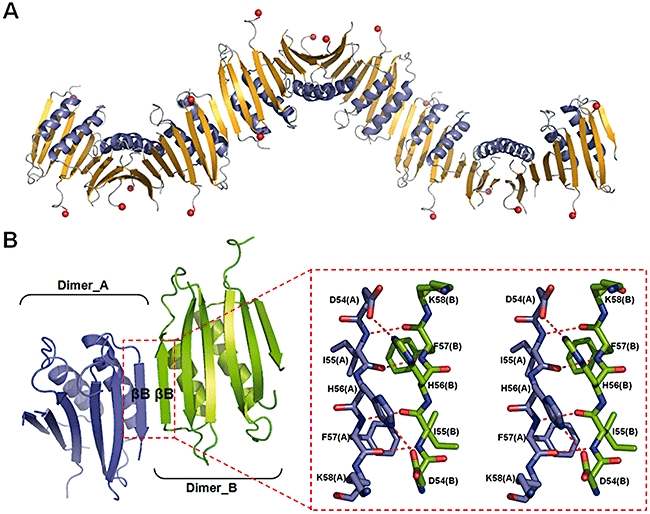
The multimeric structure of *Hp*MinE. A. Ribbon view of the multimeric structure of *Hp*MinE generated by the sixfold symmetry in the crystal. The width and length of a single turn of the multimeric structure are approximately 5.5 and 12.5 nm respectively. The N-terminus of each *Hp*MinE is shown as a red sphere. B. Close-up view of the interacting residues in the interface between two dimers (blue and green). Residues I55, H56 and F57 are involved in hydrophobic interaction, and H56 makes a hydrogen bond with D54.

Within the crystal packing, *Hp*MinE forms the spiral structure with 12 protomers per turn generated by the sixfold symmetry (Fig. 4A). The width and the length of a single turn of the spiral structure are approximately 5.5 and 12.5 nm respectively. The formation of the polymeric structure is entirely attributable to the dimer–dimer interaction mediated by the anti-parallel βB-strand. A six-stranded anti-parallel β-sheet is seen within the dimeric structure, while a 12-stranded β-sheet is seen within the tetrameric structure. The dimer–dimer interface is primarily composed of hydrophobic residues (I55, H56 and F57) (Fig. 4B). In particular, H56 of strand βB of one dimer forms a stacking interaction with H56 and hydrogen bonds with D54 of strand βB of an adjacent dimer. The surface area (∼470 Å^2^) of the interface between the two dimers is much smaller than that (∼1100 Å^2^) between two monomers. In addition, residues in strand βB are poorly conserved among Gram-negative MinE sequences (Fig. 2C). Consistent with that idea, sedimentation equilibrium experiments with purified *Ec*MinE showed that low-affinity MinE tetramers and octamers are formed *in vitro* (Zhang *et al*., 1998).

Anti-parallel β-strand interactions between MinE monomers and dimers result in anti-parallel α-helices positioned in one face (α-face in Figs 1A and 4B). The anti-parallel interaction of α-helices further stabilizes the dimer and multimer interactions by adding hydrophobic and dipole–dipole interactions between the α-helixes (Fig. S1A). The dipole–dipole interaction between two anti-parallel α-helices ranges from 12 to 24 kcal mol^−1^, which is not trivial (Fuxreiter and Simon, 2002). Thus, the anti-parallel interaction observed in the crystal structure between MinE monomers or dimers can form more stable multimeric structure than the parallel interaction. The anti-parallel interactions within and between MinE dimers position continuous α- and β-faces opposite one another. It is noteworthy that most MinE mutations affecting MinD binding localized at the dimer interface of MinE and were exposed to the β-face (*Ec*MinE mutants K19A, L22R, K19E/R21G and R10E/K11E/K12E, not I25R or D45/V49A) (Ma *et al*., 2003; Hsieh *et al*., 2010) (Fig. S1B). This implies that while the anti-MinCD domain of MinE interacts with MinD, the β-face can be located close enough to interact with MinD. For the polymer-to-polymer interaction between MinD and MinE polymers, formation of a continuous β-face through the anti-parallel interaction between MinE dimers may be more advantageous than alternative β-face exposure through parallel interactions.

### Meaning of polymeric *Hp*MinE structure observed within the crystal packing

The MinCD complex, which is associates with the inner membrane, undergoes bipolar oscillation to inhibit the cell division at polar sites. MinE is known to assemble into a ring-like structure (E-ring) near mid-cell and to undergo disassembly and then reassembly at the opposite pole of the cell. When the MinCD complex approaches mid-cell from either direction, the anti-MinCD domain of MinE interacts with the complex, causing its dissociation, which allows FtsZ ring formation at mid-cell. MinD is also known to form a polymeric structure, as evidenced by the fluorescence imaging of *E. coli* (Hu *et al*., 2002; Suefuji *et al*., 2002; Shih *et al*., 2003) and the self-organized structure forming a surface wave *in vitro* (Loose *et al*., 2008).

Polymerization and depolymerization of MinE are highly dependent on the MinD (de)polymerization process (Fu *et al*., 2001; Hu *et al*., 2002; Suefuji *et al*., 2002; Loose *et al*., 2008). Upon binding ATP, MinD directly associates with the membrane via an amphipathic helix. Upon binding to the membrane, MinD assembles into a filamentous structure that wraps around the cell cylinder. When the MinD polymer extends far enough towards mid-cell, MinE is recruited to the membrane by the membrane-bound MinD. Although MinE is present at low concentrations (∼1.4 µM) in *E. coli* cells, it can reach concentrations high enough for E-ring formation near mid-cell via recruitment to the membrane by MinD. Hsieh *et al*. (2010) recently proposed that the N-terminal anti-MinCD domain of MinE recruited to the membrane by MinD interacts directly with lipids in the membrane, especially cardiolipin, which leads to E-ring formation at mid-cell. Polymerization of MinE occurs via its TSD, and interactions with the membrane and MinD are via the N-terminal region including the anti-MinCD domain (Zhao *et al*., 1995; Ma *et al*., 2004; Hsieh *et al*., 2010). Within the crystal structure of *Hp*MinE, the anti-MinCD domain (residues 1–12 in MolA/residues 1–15 in MolB) was disordered and invisible, which implies that the fold of the newly defined anti-MinCD domain is independent of the TSD. It is therefore plausible that the anti-MinCD domain of MinE interacts with MinD or the membrane, irrespective of the multimeric state of MinE.

The N-terminal domain (residues 1–31) of *Ec*MinE reportedly interacts with helix α7 of *Ec*MinD (Ma *et al*., 2004), and mutations in helix α7 suppress the influence of MinE on the ATPase activity of MinD (Zhou *et al*., 2005), which implies the anti-MinCD domain of MinE interacts with the helix α7 region of MinD. In the modelled *Hp*MinD dimer structure, helix α7s are located at the edge of the dimer interface, possibly providing a docking surface for the interaction with the two anti-MinCD domains within the MinE dimer (Fig. S2).

To further analyse the dimer–dimer interaction observed in the crystal structure of native *Hp*MinE, we compared the crystal structures of *Hp*MinE crystallized in two different space groups. Native and Se-Met-labelled crystals of *Hp*MinE were crystallized in the P6_4_ and P6_5_ space groups respectively. The root-mean square deviation (RMSD) between the two dimers, tetramers and hexamers were 0.83, 1.41 and 2.62 Å respectively (Fig. S3). The conformation of the dimer was well conserved in the two crystal forms, but the dimer–dimer interaction began to deviate in the tetramer formation, and the packing was tilted about 24° in hexamers. This implies that: (i) the dimer interaction is stable, which is consistent with its larger surface area (22.8% of total surface area) and (ii) the supra-molecular packing is not as rigid as the dimer and has a degree of rotational freedom along the multimerization axis. This is mainly because the dimer–dimer interface formed by an anti-parallel βB-strand interaction is not stable enough to support strong tetramer formation (6.7% of total surface area). In addition, a DALI revealed that this type of anti-parallel β-strand interaction is used for multimerization in other cases as well. The *Hp*MinE dimer had high structural similarity to TOP7 (pdb id 1QYS, *Z*_score_ = 6.4, RMSD = 2.1 Å) and S-adenosylmethionine decarboxylase (AdoMetDC), which is active as a dimer (pdb id 1MSV, *Z*_score_ = 5.2, RMSD = 3.8 Å) (Fig. S4).

Lacking *in vivo* evidence, we cannot conclude that our crystal structure represents the physiological *in vivo* polymeric structure of MinE. We propose, however, that the anti-parallel βB-strand interaction forming the tetramer and higher oligomers are likely the basis for the formation MinE polymers. Consistent with that idea, we observed this interaction in two different crystal forms and found it in other crystal structures, as well. Clearly, this interaction is not just a crystallographic artefact.

In summary, we determined the crystal structure of the full-length *Hp*MinE and redefined its TSD, including strand βA, which is involved in TSD folding and is critical for the dimer formation. This novel dimeric structure of the full-length *Hp*MinE explains the earlier reported effects of mutation on cell morphology and E-ring formation. Serial dimer–dimer interactions were observed within the crystal packing, resulting in the formation of a multimeric structure. Although the mechanism underlying formation of the MinE polymer needed to make the E-ring structure during bacterial cell division is not yet known, we suggest it may reflect formation of a multimeric structure through a series of anti-parallel β-strand interactions.

## Experimental procedures

### Protein expression and purification

DNA encoding the full-length *Hp*MinE (residues 1–77) was amplified by PCR and subcloned into the NdeI and XhoI sites of the expression vector pET-28b (Novagen), which resulted in application of an N-terminal His-tag to the expressed protein. *Hp*MinE protein was expressed in *E. coli* BL21 (DE3) cultured at 20°C, after which the cells were harvested and lysed in lysis buffer containing 500 mM NaCl and 50 mM NaH_2_PO_4_ (pH 7.5). The resultant cell lysate was applied to Ni-NTA affinity chromatography column and, after washing with lysis buffer, the *Hp*MinE protein was eluted using buffer containing 250 mM imidazole, 500 mM NaCl and 50 mM NaH_2_PO_4_ (pH 7.5). The N-terminal His-tag was then removed using thrombin, and the *Hp*MinE protein was further purified by size exclusion chromatography on a Superdex 75 column (Pharmacia) equilibrated with 20 mM Tris-HCl (pH 6.0), 150 mM NaCl. The fractions containing the recombinant protein were pooled and concentrated to 20 mg ml^−1^ by ultrafiltration. Selenomethionine (Se-Met)-labelled protein was overexpressed in *E. coli* strain B834 (DE3) cultured in M9 minimal medium supplemented with Se-Met and was purified using the same protocol used for native *Hp*MinE protein.

### Crystallization

Single well-formed crystals of *Hp*MinE protein were grown at 21°C in 2 µl hanging drops containing equal volumes of protein solution (20 mg ml^−1^) and mother liquor [100 mM MES-NaOH (pH 6.5) and 26% (w/v) PEG 3350]. Crystals grew to a maximum size of 0.1 × 0.1 × 1.0 mm over a week and were cryoprotected in reservoir solution supplemented with 10% (v/v) glycerol and flash frozen under N_2_ gas at 95 K.

### Crystallographic analysis

Native data were collected at a resolution of 2.8 Å from each frozen crystal with an ADSC Quantum Q210 CCD detector at beamline 4A in the Pohang Accelerator Laboratory, Korea. The crystals belong to space group P6_4_ with unit cell dimensions of *a* = 70.8, *b* = 70.8 and *c* = 65.5 Å. All data were processed and scaled using HKL2000 (HKL Research) (Otwinowski and Minor, 1997). Multiple anomalous dispersion data sets were collected using Se-Met-labelled crystals with an ADSC Quantum 315 CCD detector on beamline BL5 at the Photon Factory, Japan. Se-Met-labelled crystals belong to space group P6_5_ with unit cell dimensions of *a* = 38.1, *b* = 38.1 and *c* = 153.5 Å. MAD phasing was carried out using the programmes SOLVE at 3.0 Å resolution, and the phases were further improved by RESOLVE (Terwilliger, 2003). Automatic model building was carried out using the programme RESOLVE, with which about 60% of the structure was modelled. Further model building was performed using the programme O (Jones *et al*., 1991), and the refinement was carried out using CNS (Brunger *et al*., 1998). The model from the MAD data was used as a starting model for the 2.8 Å native data. After rigid-body refinement and cycles of simulated annealing, a readily interpretable map was obtained, and the structure was further developed and refined. Partial hemihedral twinning was detected and treated within the programme CNS during the refinement with the twin fraction of 0.067. Many cycles of manual rebuilding using the programme O and refinement using the programme CNS resulted in a final crystallographic *R*-value of 26.2% (*R*_free_ = 29.6%). The slightly high *R* factors probably reflect the disordered regions, which were not modelled and occupying about 21.4% of the total scattering mass (residues 1–12, 61–63 and 77 in molecule A; residues 1–15 and 63–64 in molecule B). The Ramachandran plot calculated using the programme PROCHECK (Laskowski *et al*., 1993) showed no residues with torsional angles in forbidden areas: 87.0% of the residues were in the most favoured regions and 13.0% were in allowed regions. After the structural refinement was completed using P6_4_ data at a resolution of 2.8 Å, the refined structure was transported back to the P6_5_ data at a lower resolution of 3.2 Å and further refined (*R*_work_ = 27.1%, *R*_free_ = 30.3%). The data collection and refinement statistics are summarized in Table 1. Atomic coordinates from P6_4_ and P6_5_ have been deposited at the Protein Data Bank under accession code 3KU7 and 3MCD respectively.

**Table 1:** Data collection and refinement statistics.

Crystal	Native	Se-Met derivative			
X-ray source	PAL 4A	PF BL5			
Space group	P6_4_	P6_5_			
Cell dimensions, *a, b, c* (Å)	70.8, 70.8, 65.5	38.1, 38.1, 153.5			
Wavelength (Å)	1.0000	Inflection (0.9796)	Peak (0.9791)	Remote 1 (0.9833)	Remote 2 (0.9644)
Resolution (Å)	2.8 (2.8–2.85)	3.0 (3.0–3.05)	3.0 (3.0–3.05)	3.0 (3.0–3.05)	3.0 (3.0–3.05)
No. of unique reflections	4582	2582	2568	2559	2572
Mean *I*/*σ*(*I*)	17.4 (5.6)	15.6 (3.9)	15.4 (4.4)	15.9 (3.9)	14.8 (3.5)
*R*_sym_^a^ (%)	7.2 (41.5)	6.3 (56.1)	6.6 (52.8)	5.9 (50.5)	6.7 (56.0)
Data completeness (%)	99.7 (100)	98.2 (89.4)	97.9 (84.1)	98.3 (92.6)	97.5 (82.8)
Phasing and refinement statistics					
Mean FOM (50–3.0 Å)		0.60 (SOLVE)			
Overall FOM (50–3.0 Å)		0.71 (RESOLVE)			
Resolution range (Å)	50–2.8			50–3.2	
*R*_work_^b^ total (%)	26.2			27.1	
*R*_free_^c^ total (%)	29.6			30.3	
r.m.s. bond length (Å)	0.012			0.010	
r.m.s. bond angle (°)	1.8			1.9	
Average *B*-value (Å^2^)	65.4			74.9	

a *R*_sym_ = Σ|<*I*> − *I*|/Σ<*I*>.

b *R*_work_ = Σ||*F*_o_| − |*F*_c_||/Σ|*F*_o_|.

c *R*_free_ calculated with 10% of all reflections excluded from refinement stages using high resolution data.

Values in parentheses refer to the highest resolution shells.
