# Supplementary material for: Crystal structure of Helicobacter pylori MinE, a cell division topological specificity factor
Source: Mol Microbiol. 2010 Apr 27;76(5):1222–31. doi: 10.1111/j.1365-2958.2010.07160.x (PMC2883074; doi:10.1111/j.1365-2958.2010.07160.x)
Supplement: Supplementary file 1 [file mmi0076-1222-SD1.pdf]

Supporting information

Supplementary Methods

Analytical gel filtration

*Hp*MinE, *Hp*MinEΔN and *Ec*MinEΔN were expressed with N-terminal His-tags (pET28b vector), after which the His-tags were removed for analytical gel filtration analysis. Because of problems during purification when the N-terminal His-tag (pET28b vector) was used, *Ec*MinE was expressed with a C-terminal His-tag (pET21a vector) and then purified. The purified proteins (0.5 mM in 100 μl of buffer) were subjected to analytical gel filtration run at 0.5 ml/min on a Superdex 75 10/300 GL column (Pharmacia) equilibrated with 20 mM HEPES-NaOH (pH 7.0), 150 mM NaCl and 10% glycerol. *Ec*MinE, *Hp*MinE, *Ec*MinEΔN and *Hp*MinEΔN eluted at molecular masses of 30.1 kDa, 29.7 kDa, 20.6 kDa and 18.7 kDa, respectively (Table S1). Molecular weight standards [bovine serum albumin (66,000 Da), carbonic anhydrase (29,000 Da), cytochrome C (12,400 Da) and aprotinin (6,500 Da)] were used for the calibration.

Structural model building

The comparative modeling program MODELLER v8 (Sali and Blundell, 1993) was used to generate the model of the *Hp*MinD structure. Among four MinD structures with ADP or AMPPCP or without a nucleotide, *Pyrococcus furiosus* MinD (PDB code 1G3R, AMPPCP complex structure, monomer) showed the highest sequence identity (29.5% and 53.4% homology) with *Hp*MinD and was used as the template for modeling the structure of the *Hp*MinD monomer (residues 1-252). The structure of the Fe-protein from *Azotobacter vinelandii* (NifH) was previously used to generate a model of the structure of the MinD dimer (Lutkenhaus and Sundaramoorthy, 2003), and we also used the NifH dimer topology (PDB code 1N2C) to model the structure of the *Hp*MinD dimer. In addition, the disordered regions of the *Hp*MinE dimer structure (residues 1-12 in MolA/residues 1-15 in MolB) were modeled based on a secondary chemical shift analysis of *Neisseria gonorrhoeae* MinE showing the N-terminal portion of the anti-MinCD domain has an α-helix (residues 3-8 in *Hp*MinE).

Supplementary Figures

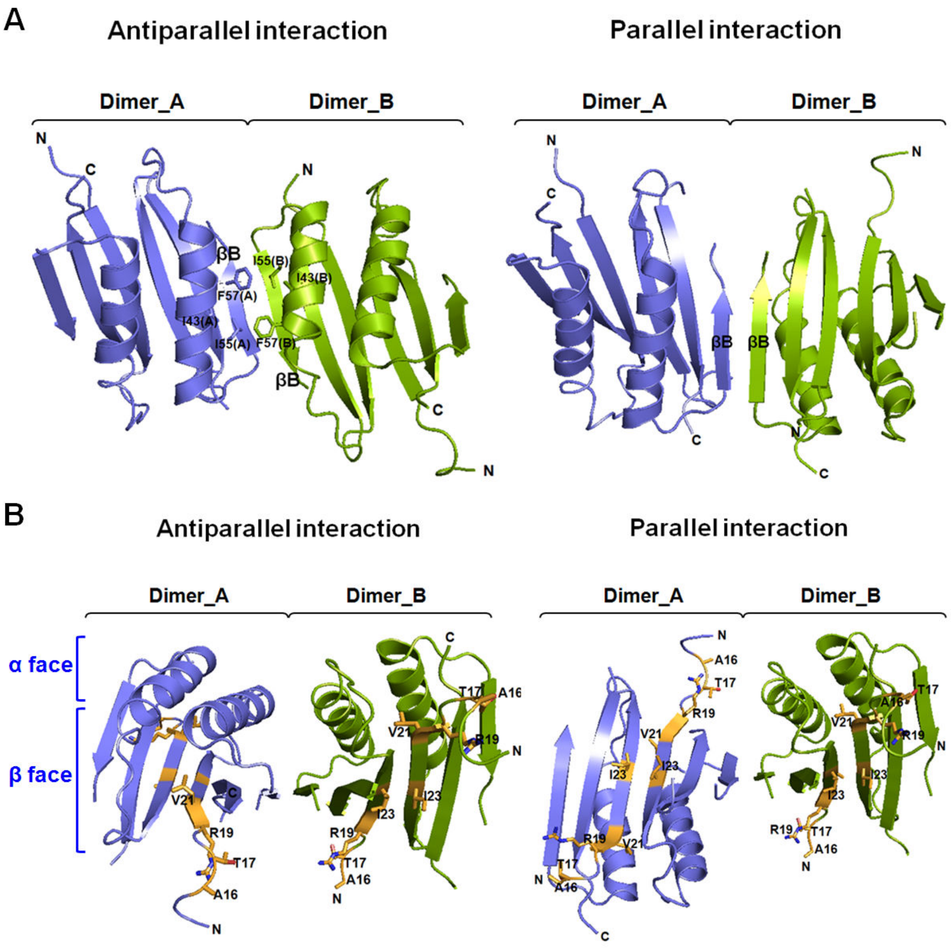

Figure S1. Comparison of antiparallel and parallel interactions within the structure of the MinE tetramer. (A) Dimer-dimer interface within the *Hp*MinE structure. (B) Representation of *Hp*MinE residues that correspond to mutated *Ec*MinE residues used to determine which residues are important for the interaction with MinD. *Ec*MinE residues R10, K11, K12, A18, K19, R21 and I25 (K10, G11, S12, A16, T17, I23, T17/R19 in *Hp*MinE) were located at the dimer interface and were exposed to the β-face; residues I25, D45 and V49 in *Ec*MinE (I23, E41 and V45 in *Hp*MinE) were not.

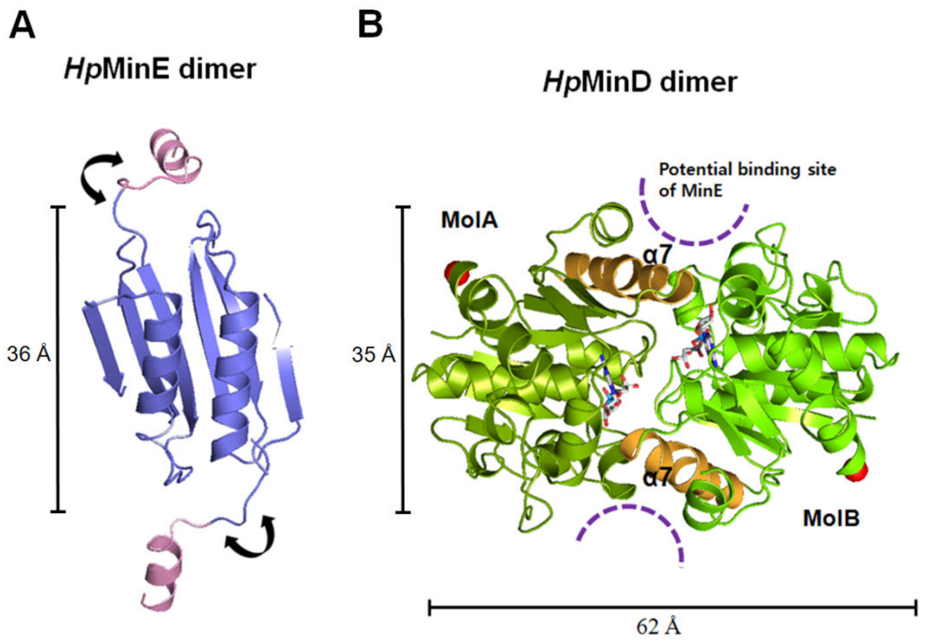

Figure S2. Molecular modeling of the *Hp*MinE anti-MinCD domain and the *Hp*MinD dimer. (A) Ribbon view showing the *Hp*MinE dimer structure, including the modeled anti-MinCD domain (magenta). Flexible linker regions between the anti-MinCD domain and the TSD are indicated in arrows. (B) Ribbon view showing the modeled *Hp*MinD dimer structure. Helix α7 (orange) of MinD is located at the edge of the dimer interface. The C-terminus of *Hp*MinD, which is responsible for interacting with the membrane, is shown as a red sphere.

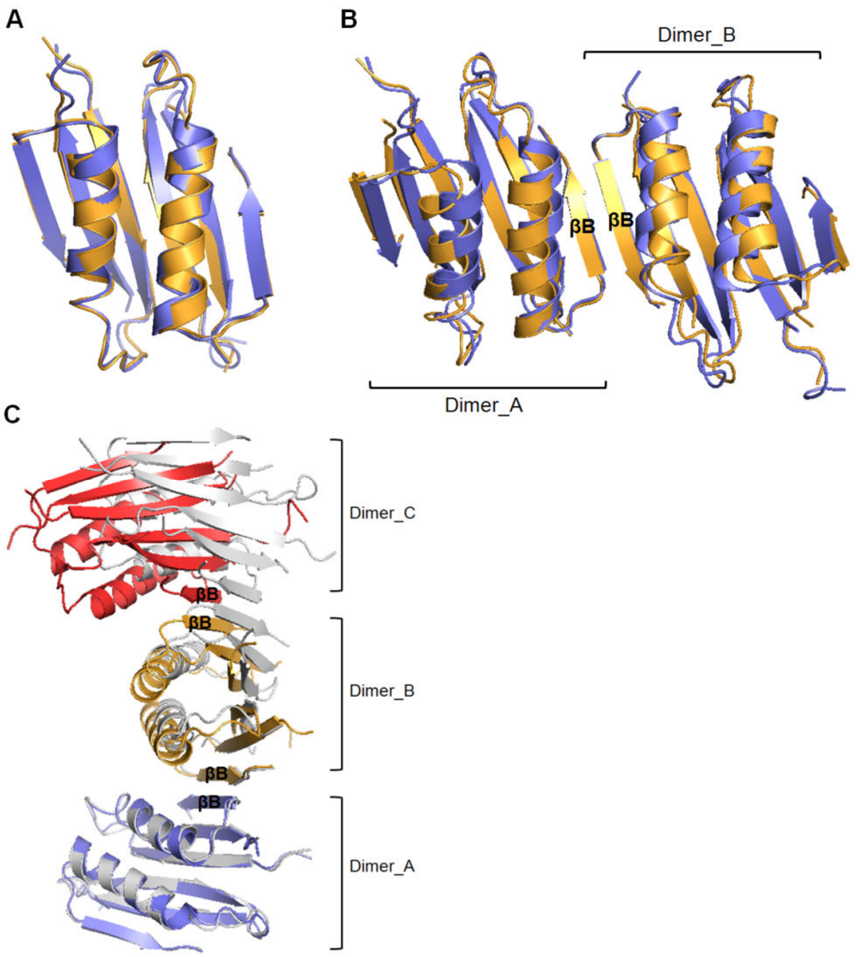

Figure S3. Comparison of the $P6_4$ and $P6_5$ structures of *Hp*MinE. (A) and (B) Superposition of the $P6_4$ and $P6_5$ structures of the *Hp*MinE dimer (A) and tetramer (B). (C) Superposition of the $P6_4$ and $P6_5$ structures of the *Hp*MinE hexamer. $P6_4$ and $P6_5$ are shown in blue and gray, respectively. The rotation angle along the multimerization axis was calculated using DynDom (Lee *et al*., 2003).

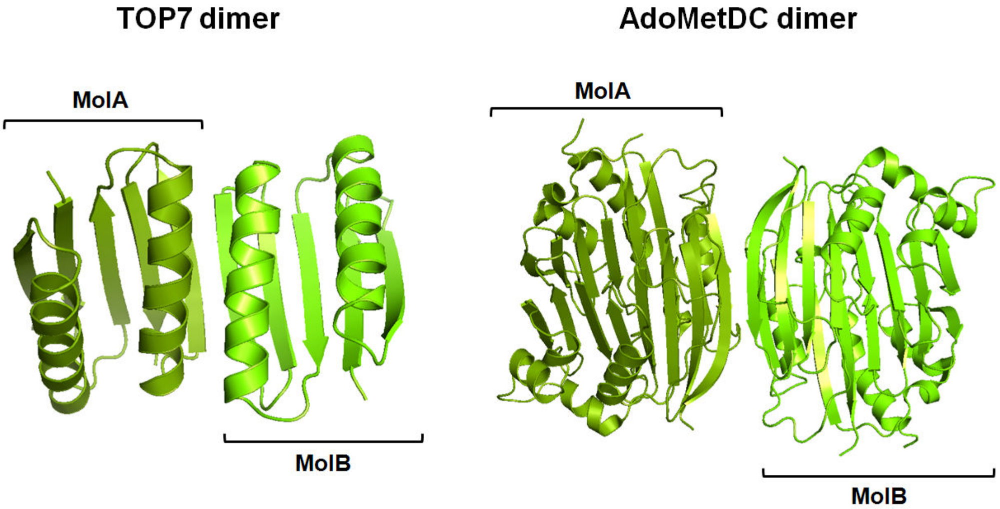

Figure S4. Structures of the TOP7 and AdoMetDC dimers. A DALI search of the MinE dimer revealed two homologous structures, TOP7 (Z_{score} = 6.4, RMSD = 2.1 Å) and S-adenosylmethionine decarboxylase (Z_{score} = 5.2, RMSD = 3.8 Å). Both of these structures form dimers through antiparallel β-strand interactions.

Supplementary Table

Table S1. Analytical gel filtration results for wild-type MinE and its deletion mutants.

	M.W. (kDa)	Superdex 75, analytical		
		Elution volume (ml)[a]	Calculated M.W. (kDa)[a]	Expected size of oligomer
*Ec*MinE (1-88)	11.3	11.82 ± 0.03	30.1 ± 0.3	2.7
*Ec*MinEΔN (32-88)	7.0	11.85 ± 0.02	29.7 ± 0.2	4.2
*Hp*MinE (1-77)	9.4	12.81 ± 0.01	20.6 ± 0.1	2.2
*Hp*MinEΔN (29-77)	6.2	13.07 ± 0.01	18.7 ± 0.1	3.0

[a]Mean ± the standard error of the mean.

Supplementary References

Lutkenhaus, J., and Sundaramoorthy, M. (2003) MinD and role of the deviant Walker A motif, dimerization and membrane binding in oscillation. *Mol Microbiol* 48: 295-303.

Sali, A., and Blundell, T.L. (1993) Comparative protein modeling by satisfaction of spatial restraints. *J Mol Biol* 234: 779-815.

Lee, R.A., Razaz, M., and Hayward, S. (2003) The DynDom database of protein domain motions. *Bioinformatics* 19: 1290-1291.
